# The Relationship between Left Ventricular Function Indices in Tissue Doppler Imaging and Exaggerated Blood Pressure Response During the Exercise Stress Test

**DOI:** 10.31661/gmj.v0i0.1323

**Published:** 2020-01-27

**Authors:** Milad Hemati, Arash Gholoobi, Ali Eshraghi, Javad Sadeghi Allah Abadi, Fereshteh Ghaderi

**Affiliations:** ^1^Atherosclerosis Prevention Research Center, School of Medicine, Mashhad University of Medical Sciences, Mashhad, Iran; ^2^Eye Research Center, Mashhad University of Medical Science, Mashhad, Iran

**Keywords:** Blood Pressure, Exercise Test, Echocardiography, Ventricular Function

## Abstract

**Background:**

Studies have indicated that exaggerated hypertension during activity and stress can be a good predictor of the incidence of hypertension. This study tries to evaluate left ventricular (LV) function by tissue Doppler to assess early changes in ventricular compliance before the onset of Hypertension (HTN) in patients with exaggerated blood pressure response during the exercise test.

**Materials and Methods:**

In this case-control study, 40 patients without a history of hypertension with systolic blood pressure less than 140/90 which referred for exercise test, were included. The exercise test was performed for all patients. Patients who had exaggerated blood pressure during the stress test were considered as cases and the controls with normal blood pressure responses. Then standard echocardiography and Tissue Doppler imaging performed and indices of LV systolic and diastolic were recorded.

**Results:**

The LV mass in cases and controls were 174.9±50.78 and 152.9±33.59, respectively (P=0.114), and LV mass index in cases and controls were 127.4±13.5 and 79.8±15.75, respectively (P=0.023). Moreover, the LV Myocardial Performance Index were 0.68±0.11 and 0.48±0.06 in cases and controls, respectively (P<0.001). The heart rate, E/A, EE, E Velocity and S velocity were measuremented. Except E/A (P=0.009), there was no significant difference between the other variables measured between the cases and controls (P>0.05).

**Conclusion:**

The results of this study showed that using 2D conventional echocardiography as a noninvasive method if performed in prestigious centers can evaluate systolic and diastolic function Tissue Doppler parameters very well in the early stages of heart disease caused by HTN.

## Introduction

The interaction between genetic factors and hemodynamic factors causes heart disease caused by HTN in patients with arterial hypertension [1,2]. The result of structural and functional adaptation leads to increased left ventricular mass (LVM), diastolic dysfunction, congestive heart failure (CHF), arrhythmia, and myocardial perfusion disorders due to dysfunction of the subendothelial vessels [3,4]. However, HTN is one of the major causes of heart failure and the mortality caused by this problem, especially when it first occurs and persists over a number of years without clinical sign and treatment, in which case life expectancy is reduced by an average of 10 – 20 years [5,6]. Untreated HTN causes excessive ventricular morphologic disorders and eventually heart failure by imposing extra load on the heart and especially the left ventricle. The most important ventricular changes in long-term hypertension are left ventricular hypertrophy [3,7,8]. It is commonly diagnosed by paraclinical instruments such as chest radiography or electrocardiography, but the best, most sensitive and precise diagnostic tool for left ventricular hypertrophy or other structural cardiac impairment is echocardiography [9-11]. It has also been well documented that the structural and functional abnormalities of the heart in primary blood pressure increase the risk of mortality and morbidity [12]. However, there is no clear relationship between the known blood pressure in the clinic and the level of structural and functional compliance of the left ventricle [1,3,6,10,12]. Reduced systolic function of the left ventricle is due to initial hypertension in the final stages of the disease. On the other hand, changes in the diastolic function of the left ventricle and hypertrophy occur in the early stages of disease [1,3,13,14]. According to studies, changes in blood pressure are directly related to physical and mental activity, and it has been reported that cardiovascular hyperactivity plays an important role in cardiovascular diseases in response to mental and physical stress. Therefore, early diagnosis of HTN is very important [3,15,16,17]. In several studies, it has been shown that exaggerated hypertension during exercise and stress can be a good predictor of future incidence of HTN [18-21]. Accordingly, exaggerated hypertension during exercise test has been defined to increase the systolic blood pressure from rest to maximum activity (≥220 mmHg) in the peak of secondary activity, and diastolic changes from rest to activity (≥15 mmhg) [18,19,20]. Considering the fact that few studies have been conducted to investigate the relationship between echocardiographic findings in patients with exaggerated hypertension during exercise test, and considering that higher incidence of hypertension was not reported in these patients in some recent studies, this study aims to evaluate left ventricular function using Tissue Doppler Imaging to analyze the early changes in ventricular abnormalities before HTN in these patients and compare them with those without HTN.

## Materials and Methods

###  Patient Selection

 The present study is an analytical and descriptive case-control study with the aim of investigating the initial changes of ventricular abnormalities before HTN in these patients and compare them with non-HTN subjects. The entrance of samples to this study was randomized. In this study, all patients admitted to the Exercise Test Department of Ghaem and Imam Reza Hospitals (general and central Hospitals in Mashhad city, Iran) were studied. All patients were carefully evaluated and examined at the time of referral. Patients with systolic blood pressure between 120 and 139 and diastolic blood pressure between 75 and 89 were included in the study. Patients who had a history of hypertension, taking antihypertensive drugs, initial systolic blood pressure above 140, or diastolic blood pressure above 90, diabetes, positive exercise test, and coronary artery disease were excluded. Written informed consent were obtained from all patients before entering the study. This study approved by Ethics Committee of Mashhad University of Medical Sciences.

###  Sample Size

 According to the preliminary criteria for the study, 410 patients were tested for exercise. The exercise test results were interpreted in these specimens. After interpreting the results, 50 patients were excluded from the study due to positive test results. Also, 340 patients were excluded due to their history of hypertension, cad, and dissatisfaction with the study. Finally, 20 patients with exaggerated blood pressure during exercise as a case group were studied. In addition, 20 healthy subjects without any exaggerated blood pressure during exercise as a control group were randomly enrolled and studied.

###  Study Protocol

 Exercise test (Stress test system ast-3000, Avicenna co, Iran) was performed for all subjects. Patients who had exaggerated blood pressure during exercise test were considered as case group and samples with normal blood pressure during exercise test were considered as a control group. In samples with a history of smoking, it was recommended that the patient quit smoking at least 6 hours before the exercise test. The Brouce protocol was used in the exercise test and systolic and diastolic blood pressure were measured during the last minute of each exercise as well as in the peak test. The patients continued the test until they reached the target (heart rate based on age-220), and if the patient had a systolic blood pressure of 200 mmHg or above or a diastolic blood pressure of 15 mmHg higher than the base diastolic pressure, it was considered as exaggerated blood pressure during exercise. All equipment used in this study were calibrated. After identification, patients underwent Tissue Doppler Imaging and standard 2D conventional echocardiography (Vivid 7 expert, USA), and the left ventricular systolic and diastolic indexes and other information about age, sex, height, weight, and BMI of patients were recorded in the relevant checklists.

###  Ethical Statement

 After entering the samples in this study, they were all taken to participate in the study of informed consent. The Ethics Committee approval of Mashhad University of Medical Sciences was obtained for this study. The code of ethical approval is IR.MUMS.fm.REC.1394.333.

###  Statistical Analysis

 After collected of information, results were evaluated by SPSS13 software (SPSS Inc., Chicago, Illinois, USA). Descriptive data were collected from the frequency distribution table, central indexes, distribution and percentages. Continuous quantitative data were compared between the two groups using the independent t-test and Chi-Square test was used to compare the discrete data between the groups. In addition, the correlations between variatans were performed and 0.05 was considered as the significance level.

## Results

 This study was performed on 40 samples who were referred to Ghaem and Imam Reza hospitals in Mashhad to do exercise tests. The 20 samples were randomly assigned to each of the case and control groups. After performing the exercise test and recording the required data, standard 2D conventional echocardiography and Tissue Doppler Imaging were performed for all patients. The 21 (52.5%) patients in this study were male and the rest were female. The mean age of patients in cases and control groups were 44.05±9.65 and 43.02±7.4 years, respectively. There was no significant difference between the two groups in terms of age and gender (P>0.05). Three (7.5%) patients had a history of smoking, and seven (17.5%) had a history of hyperlipoproteinemia (HLP). The mean BMI of patients was 26.6 (Table-1). There was no significant difference between systolic and diastolic blood pressure and heart rate at the beginning of the study in both groups (P>0.05). Echocardiographic findings showed that the mean LVM in the case group was 174.9±50.78 and in the control group was 152.9±33.59, which did not show a significant difference between the two groups (P=0.114). Mean LVM index, however, showed a significant difference between the two groups (127.45±13.01 and 79.8±15.75 gr/cm2, respectively) (P=0.023). The mean left ventricular Myocardial Performance Index (LV MPI) in the case group was 0.58±0.11 and in the control group was 0.38±0.06, which was statistically significant between the two groups (P<0.001). In addition, E/A in case and control groups was 6.81±18.27 and 1.08±0.17, respectively (P=0.009). The EE and DT factors were used to assess the rate of atrial filling pressure. EE in case and control groups was 29.74±13.27 and 8.0±1.21 (P=0.201), and DT was 174.35±59.13 and 196.1±19.63 milliseconds, respectively (P=0.779). The Ejection fraction in the case group was 58.7±11.74 percent and in the control group was 57.5±6.09 percent (P=0.335). The E Velocity propagation (E/Vp) was used to study the fluctuating flow through mitral in the initial contraction phase, which was lower than that of the control group (P<0.001). S velocity was used to evaluate the left ventricular function during systolic injection. There was no significant difference in the group (P=0.495) (Table-2). Investigating the correlation between age and 2D conventional echocardiography findings in the case group showed that E/A with correlation coefficient of -0.52 and P = 0.019 had a significant reverse relationship with age, while E/Vp with correlation coefficient of 0.52 and p = 0.001 had a direct relationship with age. On the other hand, the left ventricular mass and DT with correlation coefficient of 0.344 and 0.272, respectively, had a direct relationship with age. In addition, the correlation between systolic blood pressure after exercise test and echocardiographic findings in the case group showed that LV MPI with correlation coefficient of 0.647, P<0.001 and E/Vp with correlation coefficient of 0.623 and P<0.001 had significant positive correlation with systolic blood pressure after exercise tests. E/A with correlation coefficient of -0.268 had the highest inverse relationship with systolic blood pressure (Table-3).

## Discussion

The results of this study showed that transthoracic 2D conventional echocardiography, especially Tissue Doppler Imaging, can detect diastolic and systolic changes in patients with exaggerated blood pressure responses. Echocardiography is the primary diagnostic method for left ventricular dysfunction [22,23]. This method can examine the size of the cavities, ventricular contractility, valve function, and blood flow using Doppler. The size of the ventricular chamber can be measured directly by calculating the ejection fraction. The flow rate and direction in the valve paths can be measured using Doppler imaging and allows the calculation of pulmonary arterial pressure and cardiac output [11,24]. In several studies, it has been shown that exaggerated hypertension during exercise and stress can be a good predictor of future HTN incidence [3,15,16]. Therefore, considering that quantitative studies have been conducted to investigate the relationship between echocardiographic findings in patients with exaggerated hypertension during exercise testing, this study aims to evaluate left ventricular function using Tissue Doppler Imaging in order to investigate the early changes in ventricular abnormalities before the onset of HTN in these patients and comparing them with healthy subjects. The results of this study showed that there was no significant relationship between demographic characteristics in the case and control groups. Furthermore, there was no significant difference between systolic and diastolic blood pressure and heart rate between the case and control groups at the beginning of the study. The study of Herkenhoff *et al*. *et al*. [25] confirms these results, but the results of the study by Mantero *et al*. [26], in examining the relationship between heart rate echocardiography indexes, showed that heart rate had a significant effect on echocardiographic indexes. Therefore, when diastolic parameters of left ventricular function are evaluated, it is important to note the heart rate. In addition, the results of our study showed that LVM and LVM index factors in the case group were higher than the control group. In a study by Dunn *et al*. [27], it was stated that in the comparison of the three groups of patients; the first group with normal ECG and CXR, the second group with left atrium abnormality in normal ECG and CXR and the third group with LVH in ECG or CXR or both, LVM increased significantly and progressively from group one to two and from two to three. Similarly, in the study of Al-Abasi *et al*. [28], systolic blood pressure in resting condition was positively associated with LV mass. The wall thickness also had a positive correlation with the response of systolic blood pressure to stress, but the relationship between LVM and the pressure response was often non-significant. Moreover, in the study of Markovitz *et al*. [29], which was performed on exaggerated blood response to laboratory stress tests, there was a small correlation between BP response to laboratory stress and LV mass. These results indicate that there is a relationship between exaggerated hypertension in the exercise test, LVM and LVM index. In addition, the results of our study showed that mean LV MPI and E / A in the case group were significantly higher than the control group. The study of Manteroa *et al*. [26] and the study of Mabarotic *et al*. found that E / A levels increased significantly in patients with increased BP. Marabotti *et al*. [30] also reported that patients with borderline hypertension had higher posterior wall and interventricular septum thickness compared with normal group and lower than the hypertension group. Furthermore, with respect to diastolic indexes, this group had more A Peaks and A/E ratios than the control group. But this group did not have significant difference with proven hypertension group. In addition, the results of our study showed that there was no significant difference between Ejection fraction and S velocity in the case and control groups, but E/Vp was significantly higher in the control group. In a study by Marabotti *et al*., patients with borderline hypertension had a lower ejection fraction than the rest of the subjects. But unlike our findings, in the study of Dunn *et al*. [27], a significant EF decline was observed in subjects with left atrium abnormalities in normal ECG and CXR. In investigation of the correlation between systolic blood pressure after exercise test and echocardiographic findings in the case group, LV MPI and E/Vp factors had a significant positive correlation with systolic blood pressure after exercise test. Therefore, it seems that further studies are needed on these two variables. Moreover, the results of this study showed a reverse correlation between age and blood pressure-based factors. By increasing age, exaggerated hypertension increases. In the study of Mantero *et al*. [26], on the association between echocardiography indexes and age, it was shown that age affects the peak speeds of wave A and E, E / A ratio, wave A integral, E/A ratio integral, primary and secondly filling ratio, E wave drop and time of drop. E / A ratio inversion was also observed in people over 70 years of age. Multivariate analysis also confirmed that age has an important effect on left ventricular diastolic indexes. One of the main constraints in the study was the limited number of patients with an exaggerated blood pressure response. Due to the limited number of patients with hypertension and also lack of previous history, we tried to collected samples in two central hospitals in Mashhad. Due to the specialty of the echo in this study and the free of charge test, due to the time of the test, some patients did not agree to enter the study. Therefore, according to a study by GROSSMAN *et al*. in 2014, sample size of our study, 40 patients were determined [31].

## Conclusion

 The results of this study showed that transthoracic 2D conventional echocardiography, especially Tissue Doppler Imaging, as a non-invasive method, can detect diastolic and systolic changes in patients with exaggerated blood pressure responses in the exercise test prior to the onset of HTN, and can detect systolic and diastolic Tissue Doppler parameters of the systolic and diastolic function of the ventricle in the early stages of heart disease caused by hypertension. In addition, our study shows disorders in diastolic function of patients with hypertension in exercise tests compared to controls, which can indicate the incidence of HTN in these individuals in the future.

## Acknowledgement

 This work was financially supported by the Mashhad University of Medical sciences, Mashhad, Iran (grant number is 940206). The present study extracted from the thesis of Dr. Milad hemati, Resident of Cardiology, atherosclerosis prevention research center, Mashhad University of medical science, Mashhad, Iran.

## Conﬂict of Interest

 The author(s) indicated no conﬂicts of interest.

**Table 1:** The Demographic Characters in Cases and Controls

**Variation**		**Cases** **n=20**	**Controls** **n=20**	**Total** **n=40**	**p- value**
**Sex**	Male	13(65%)	8(40%)	21(47.5%)	0.102
	Female	7(35%)	11(60%)	18(52.5%)	
**Age**		44.05±9.65	43.02±7.4	43.53±8.59	0.06
**Weight**		77.28±9.27	79.32±6.78	78.3±8.13	0.082
**Height**		170.42±5.7	169.78±4.98	170.24±5.44	0.8
**BMI**		26.00±2.74	27.12±3.36	27.08±3.1	0.502
**Smoking**		3 (15%)	1 (1.25)	4(10%)	0.115
**HLP**		5(25%)	2(17.5%)	7(17.5%)	0.204

**Table 2:** Evaluate of 2D Conventional Echocardiography Finding in Cases and Controls

**Variation**	**Cases**	**Controls**	**P-value**
**Before exercise test SBP**	123.75±10.37	119±3.07	0.165
**After exercise test SBP**	197.25±15.17	128.5±6.9	0.134
**Before exercise test DBP**	75.78±7.68	79±3.7	<0.001
**After exercise test DBP**	92.36±10.05	84±4.47	0.002
**Heart rate**	68.3±3.13	65.1±6.11	0.203
**LV mass**	174.9±50.78	152.9±33.59	0.114
**LVM index**	127.45±13.01	79.8±15.75	0.023
**LV MPI**	0.58±0.11	0.38±0.06	<0.001
**BSA**	1.8±0.18	1.89±0.05	0.044
**LA volume index**	18.52±4.75	17.13±4.48	0.347
**E/E**	13.27±29.74	8±1.21	0.201
**DT**	174.35±59.13	196.1±19.63	0.779
**EF**	58.7±11.74	57.5±6.09	0.335
**E/A**	6.81±18.27	1.08±0.17	0.009
**E/ Vp (cm/s)**	51.7±0.08	60.7±0.39	<0.001
**S velocity**	7.7±1.78	7.3±0.8	0.495

**Table 3:** Correlation Between Age and Systolic Blood Pressure after Exercise Test Versus Echocardiographic Findings in Cases

**Variate**	**Systolic pressure**		**Age**	
	**Correlation**	**P-value**	**Correlation**	**P-value**
**S velocity**	0.078	0.631	-0.089	0.708
**LV MPI**	0.647	<0.001	-0.018	0.939
**LV mass**	0.245	0.127	0.344	0.138
**EA**	-0.268	0.094	-0.52	0.019
**DT**	0.012	0.94	0.272	0.245
**E/ Vp**	0.623	<0.001	0.52	0.001
**LVM index**	0.231	0.151	0.1	0.675
